# Measuring vaccine effectiveness from limited public health datasets: Framework and estimates from India’s second COVID wave

**DOI:** 10.1126/sciadv.abn4274

**Published:** 2022-05-06

**Authors:** Abhiroop Mukherjee, George Panayotov, Rik Sen, Harsha Dutta, Pulak Ghosh

**Affiliations:** 1 The Hong Kong University of Science and Technology, Clear Water Bay, Hong Kong.; 2 University of New South Wales, Sydney, NSW, Australia.; 3 Indian Institute of Management, Bangalore, India.

## Abstract

Despite an urgent need, authorities in many countries are struggling to track COVID vaccine effectiveness (VE) because standard VE measures cannot be calculated from their public health data. Here, we use regression discontinuity design (RDD) to estimate VE, motivated by such limitations in public health records from West Bengal, India. These data cover 8,755,414 COVID vaccinations (90% ChAdOx1 NCov-19, almost all first doses, until May 2021), 8,179,635 tests, and 141,800 hospitalizations. The standard RDD exploits age-based vaccine eligibility; we also introduce a new RDD-based VE measure that improves on the standard one when better data are available. Applying these measures, we find a VE of 55.2% (95% confidence interval: 44.5 to 65.0%) against symptomatic disease, 80.1% (63.3 to 88.8%) against hospitalizations, and 85.5% (24.8 to 99.2%) against intensive care/critical care/high dependency admissions or deaths. Other data-deficient countries with age-based eligibility for any vaccine—and not just COVID vaccines—can also use these easy-to-implement measures to inform their own immunization policies.

## Teaser

VE can be calculated by exploiting age-based eligibility, even when data limitations preclude using standard methods.

## INTRODUCTION

Vaccine effectiveness (VE) estimates obtained using public health datasets have important advantages. First, such datasets cover a wide range of people, alleviating concerns that differences in serostatus (*1*–*3*) across populations can limit the usefulness of estimates that rely only on specific populations within countries. For example, a test-negative design (TND) or cohort study using health care workers—who are more likely to have previous infection–induced immunity (*4*)—may not provide valid VE estimates for the general population. Second, public health datasets are typically large, allowing health authorities to estimate VE against rare but severe outcomes [intensive care unit (ICU) admissions or deaths]. Such estimates are especially important in the current Omicron-driven stage of the coronavirus disease (COVID) pandemic, with many countries choosing to “live with the virus,” as long as their vaccinated populations are spared from critical outcomes (*5*). Last, VE estimates using population-wide public health data can also help address vaccine hesitancy by assuring people that the vaccine has worked well for others like them (*6*).

However, public health datasets in most low/middle-income countries (LMICs) do not contain adequate information to calculate VE using standard methods like TND (*7*, *8*). In particular, vaccination status information is often not recorded for people who took COVID tests. As a result, almost all large-scale evidence to date on the real-world effectiveness of vaccines comes from developed countries. For example, view-hub.org, a comprehensive database of 181 COVID VE studies (as of 10 February 2022), contains only 12 from LMICs—these are all cohort studies or TNDs (hence, the vaccination status of all participants is known in each case) and only three are based on large-scale public health data (*9*–*11*). The general lack of VE information for individual LMICs hinders the formulation of essential health policies in these countries. For example, their health authorities may wonder whether it is optimal to inoculate more people with a first dose or to provide enhanced protection by administering multiple doses to a few. This uncertainty arises because, on the one hand, evidence from developed countries shows reduced VE against the Delta and Omicron variants that might make partial vaccinations ineffective [e.g., (*12*)]. On the other hand, a higher infection-induced immunity might lead to a higher first-dose VE in these LMICs than what has been found in developed countries—this would make it optimal to use a partial vaccination strategy and provide wider protection early on, especially given vaccine scarcity (*13*–*15*).

Here, we demonstrate, using public health data from West Bengal, that VE can be estimated through a regression discontinuity design [RDD; see (*16*–*18*)] even when alternative VE measures cannot be calculated. We use two RDD-based VE measures, both of which rely on age-based discontinuities in vaccinations (e.g., when West Bengal started vaccinations, only those aged 45 years or older were eligible—and hence much more likely to be vaccinated than those aged 44 or below). The first measure, which we call “standard RDD,” is similar to what has been used in prior VE studies [including those on COVID in developed countries, e.g., (*19*, *20*)] and can be calculated when vaccination information is not available for outcomes (as is the case with COVID testing data in West Bengal).

However, when information on the vaccination status for COVID outcomes is available (as is the case with COVID hospitalizations in West Bengal), we propose a second measure, which we call “breakthrough-based RDD” (B-RDD). This measure is novel in the VE literature; it uses the difference in breakthrough hospitalizations that the standard RDD does not use. As we show in Methods, this allows the B-RDD to recover the correct VE under milder assumptions than the standard RDD; hence, it can help provide more robust VE estimates in RDD-based studies that have access to linked data.

Moreover, both these measures can avoid bias arising from (i) the data being noisily measured (particularly likely when health systems are overwhelmed) and (ii) unmeasured confounding factors (*16*, *19*). Besides, they can be implemented using a spreadsheet, which makes them easy to use by public health officials (see spreadsheet S1).

We apply these measures to calculate the effectiveness of COVID vaccines against the Delta variant using a large but limited public health dataset from West Bengal, India. Other researchers can apply these measures to estimate the effectiveness of existing or future vaccines against Omicron or newer COVID variants as long as there are age-based eligibility criteria [e.g., see (*21*) for various countries]. Going beyond COVID, since most countries use such eligibility criteria for various other vaccination programs, these measures can, in general, be useful additions to the public health toolkit, especially in data-deficient countries.

## RESULTS

### Age discontinuity in West Bengal vaccinations

Vaccinations in West Bengal began in January 2021, and first targeted health care and frontline workers. On 1 March 2021, eligibility was extended to people with comorbidities aged 45 years or older, and to everyone aged 60 or older. It was further extended to everyone aged 45 or older on 1 April 2021, and lastly, to all adults (18 and older) on 1 May 2021. Thus, we use vaccination information from 1 March 2021 (when the 45-year age cutoff came into effect) until 30 April 2021 [when those younger than 45 were also made eligible, diluting the difference across the cutoff that is key for the RDD; see, e.g., (*19*)]. COVID vaccination required providers to verify a person’s age against an officially issued form of identification, such as a person’s Aadhaar card. Given that most identification cards were registered well before vaccine eligibility was determined, manipulating age for eligibility purposes was virtually impossible.

**Fig. 1.**
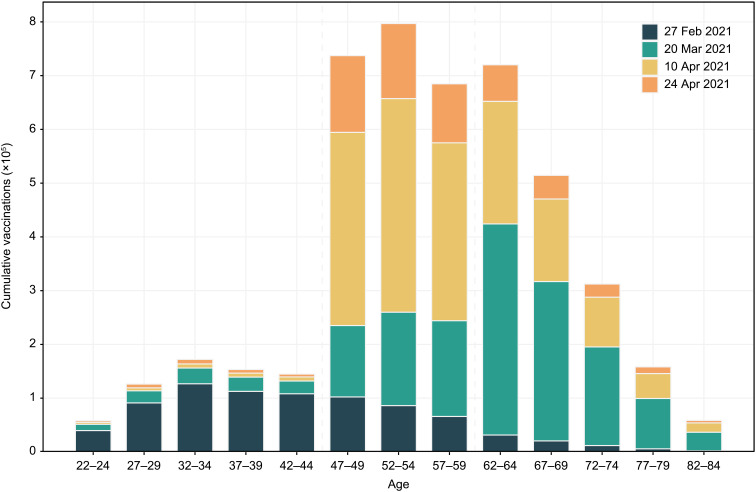
Cumulative vaccinations. This figure shows the number of people with first dose vaccinations in West Bengal (*y* axis), for each age group shown on the *x* axis. At each of the reported four dates, the cumulative vaccinations equal the sum of the bars corresponding to this date and all previous dates (if any). We drop ages that end at zero, one, five, and six to account for the pronounced age heaping in our data (see below for details on age heaping).

Using our vaccination data, we verify in Fig. 1 that the official cutoff was enforced, i.e., a substantially higher number of people just above the age of 45 years were vaccinated than those just below that age [more than 90% of vaccinations were ChAdOx1 NCov-19, and the rest are BBV152 (*22*)]—satisfying a key requirement for the applicability of RDD. The difference persistently increased before 1 May, i.e., when those below the age of 45 were still not eligible for vaccination. Note that there is no evidence of a discontinuity at age 60, and therefore, we cannot use that cutoff here.

We estimate VE for those who were administered at least one dose of either vaccine given in West Bengal. This is due to two limitations of our data. First, information on second versus first dose, including the respective dates of vaccination, is missing for 36% of those who were reported by their hospitals as having been administered a vaccine. Second, we cannot differentiate between the effectiveness of the two types of vaccines administered in West Bengal—our aggregate vaccination data do not contain this information, while detailed vaccination data from hospitals, again, are missing for many who were vaccinated. We assume that vaccines provide protection 2 weeks after the first dose, following the World Health Organization guidance [page viii in (*7*)].

### Vaccine effectiveness analysis

The VE results in this section are based on the two RDD-based measures discussed earlier. The methodological framework underlying these measures and the data used to implement them are described in Methods.

**Fig. 2.**
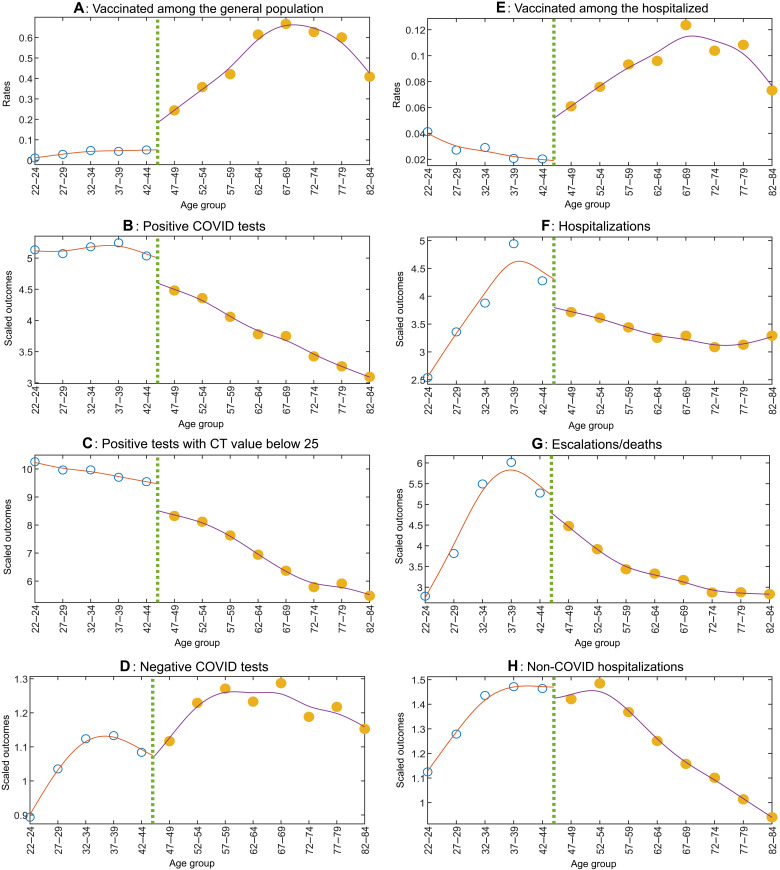
Vaccinations and outcomes across age groups. Each dot in the plots represents data aggregated over the three consecutive ages shown on the *x* axis, and a vertical line in each plot marks the cutoff age of 45 years. The curves in each plot are obtained by fitting separate smoothing splines (with a smoothing parameter of 0.05) through the dots on the left and right of the cutoff age of 45 years. (**A**) Weighted average vaccination rates in the population for each age group. (**B**) The number of RT-PCR–positive COVID infections averaged across each age group in the postvaccination period (13 March 2021 to 14 May 2021), scaled by its corresponding preperiod value (5 September 2020 to 12 March 2021). (**C** and **D**) The same ratios for infections with high viral load [cycle threshold (CT) Egene/Ngene value < 25] and RT-PCR–negative COVID tests, respectively. Similarly, (**E**) shows vaccination rates among those who were hospitalized, and (**F**) to (**H**) show hospitalizations, escalated outcomes, i.e., ICU/critical care unit/high dependency unit admissions or deaths, and non-COVID hospitalizations, all scaled as above. The postvaccination period for the hospitalization sample in (E) to (H) starts on 10 April 2021 due to incomplete data on vaccination status of those hospitalized; the prevaccination period is as before. Details on the calculation of these variables are described in the section “Calculating VE in our sample.”

First, note that the various COVID outcomes in Fig. 2 (B, C, F, and G) exhibit clear discontinuities at the same 45-year age cutoff as do the vaccinations (in Fig. 2, A and E). We find no such discontinuity when we examine those who tested negative for COVID in Fig. 2D or those hospitalized for non-COVID reasons [and tested reverse transcription polymerase chain reaction (RT-PCR)–negative in hospital] in Fig. 2H. This attenuates concerns that other reasons, as for example access to testing or hospitals, may be driving the discontinuity in COVID outcomes.

The proportional drop in COVID outcomes at the cutoff—a key input for both RDD-based VE measures—can be calculated from the points corresponding to the 42-44 versus 47-49 age groups in Fig. 2. The population vaccination rates corresponding to the same age groups, required for the standard RDD, are presented in Fig. 2A. The proportion vaccinated among those hospitalized in these age groups, required for the B-RDD, is presented in Fig. 2E. We show these inputs and resultant VE estimates in Table 1 under Local randomization (which assumes that individuals in the two age groups have been randomly picked from the same pool, i.e., they are interchangeable).

**Table 1.** Vaccine effectiveness estimates. This table shows the inputs to the two VE formulas from Eqs. 1 and 2, with VE estimates and 95% bootstrap confidence intervals. Under “Local randomization,” these inputs correspond to the age groups nearest to the 45-year age cutoff. In panel A, the VEs are calculated with the standard RDD (1), and in panel B, they are calculated with the B-RDD (2). Under a “Continuity-based framework,” the inputs to the formulas are obtained from the points in Fig. 4 where the smoothing splines intersect the vertical line at the cutoff. “Tests with CT value ***<*** 25” refers to symptomatic COVID infections with high viral load (cycle threshold Egene/Ngene value ***<*** 25), and “Escalations/deaths” refers to escalated outcomes such as ICU/critical care unit/high dependency unit admissions or deaths. CT, cycle threshold.

	**Local randomization**					**Continuity-based framework**				
**Panel A**	***v_L_***	***v_R_***	***X***	**VE**	**95% CI**	***v_L_***	***v_R_***	***X***	**VE**	**95% CI**
RT-PCR–positive COVID tests	5.0	24.4	11.0	55.2	[44.5, 65.0]	5.1	18.4	8.1	59.1	[38.4, 78.7]
Tests with CT value < 25	5.0	24.4	12.8	64.0	[33.7, 91.1]	5.1	18.4	10.1	73.5	[13.1, 126.2]
**Panel B**	**μ** ***_L_***	**μ** ***_R_***	***X***	**VE**	**95% CI**	**μ** ***_L_***	**μ** ***_R_***	***X***	**VE**	**95% CI**
Hospitalizations	2.0	6.1	13.1	80.1	[63.3, 88.8]	1.9	5.2	11.9	81.8	[53.5, 92.9]
Escalations/deaths	1.4	4.7	15.1	85.5	[24.8, 99.2]	1.5	3.6	8.3	82.6	[−88.8, 212.3]

All these inputs can also be calculated under a Continuity-based framework [e.g., as in (*23*)]—it provides asymptotically unbiased VE estimates even if Local randomization does not hold, as long as the average outcomes change smoothly with age, absent vaccinations. Under this framework, we calculate the above inputs from the points where the smoothing splines in Fig. 2 intersect the vertical line representing the cutoff and also show them in Table 1. The resultant VE estimates are similar to those obtained under Local randomization.

Note that our VE estimates increase with disease severity. Furthermore, these VE estimates are weighted averages of the VEs for two doses. Table 2 shows estimates for lower and upper bounds of first-dose VE only, calculated using methods described in the Supplementary Materials.

**Table 2.** Bounds for first dose vaccine effectiveness. This table shows, for four different outcomes, VE bounds for the first dose only. These bounds are calculated following the procedure in Supplementary Materials (“Estimating VE for a two-dose regime”). All numbers are shown in percentage.

	**Left**	**Right**	**Difference**	**Weight**
Vaccination rates first dose	5.0	24.4	19.4	95.8
Vaccination rates second dose	3.1	3.9	0.9	4.2
	**Upper**	**Lower**	**Lower (×3)**	**Lower (×5)**
RT-PCR–positive COVID tests	55.2	53.2	48.7	43.1
Tests with CT < 25	64.0	62.4	58.7	54.3
Hospitalization	80.1	79.2	77.2	74.7
Escalations/deaths	85.5	84.9	83.4	81.6

Moreover, even if the vaccination status is available, we can still use the standard RDD to calculate VE against hospitalizations. For example, we obtain a VE of 67.5% (compared to 80.1% with B-RDD) for hospitalizations. This difference in estimates reiterates that the bias in the standard RDD measure that we discuss in Methods can be material. Note also that, here, we estimate one VE number for our sample period, although one could also use COVID outcomes from different time periods—if available—to calculate a time series of VE estimates using the same methodology.

## DISCUSSION

**Fig. 3.**
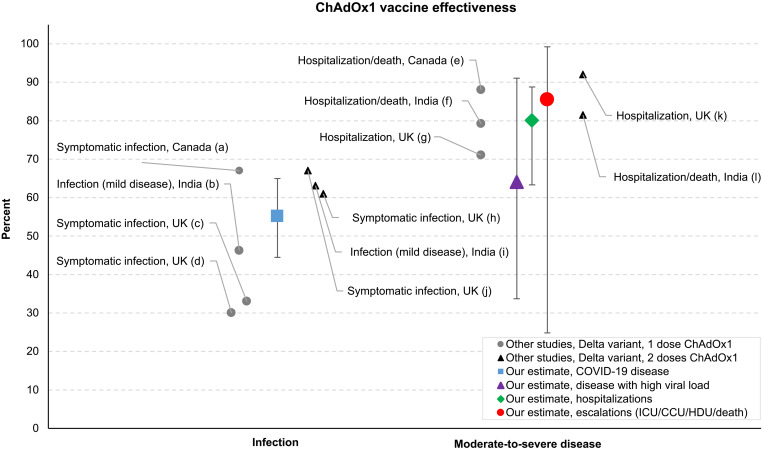
Comparisons. Our VE estimates are compared to those from several other studies on the VE of ChAdOx1 NCov-19 against the Delta variant. Comparisons are made with respect to (i) outcome (infection or hospitalization) and (ii) the number of doses administered. The figure also shows the country where the respective VE was obtained. The numbers used for comparison are sourced as follows: (a) and (e): table 3 in (*24*); (b) and (i): table 2 in (*27*); (c) and (h): table S5 in (*26*); (d) and (j): table 2 in (*12*); (f) and (l): table 3 in (*27*); (g) and (k): table 1 in (*36*). CCU, critical care unit; HCU, high dependency unit.

Figure 3 compares our VE numbers against other studies on VE for ChAdOx1 NCov-19 against the Delta variant (or in settings where the Delta variant was thought to be prevalent). We refer to studies that report results for both the first and second doses. Given that an overwhelming majority of observations in our data are on first doses only, the VE of the first dose is the more relevant benchmark for our estimates (this is corroborated by the first-dose-only VE bounds in Table 2 being close to the baseline estimates in Table 1). Furthermore, since 90% of the vaccinated population in West Bengal took the ChAdOx1 NCov-19, we refer only to studies on this vaccine. We also recognize that VE can depend on age, the waning of vaccine-induced immunity over time, differences across recording systems for outcomes, and other factors, and hence comparisons of VE estimates across studies should be interpreted with caution.

Given these caveats, and as shown in Fig. 3, our numbers are lower than those reported in (*24*) for Canada (VE of 67% against symptomatic infections and 88% against hospitalization or deaths) for the first dose. Our VE against hospitalization is similar to (*25*), which report VE values of 71% for the first dose and 92% for the second dose, and is substantially higher than those reported in appendix table S5 in (*26*), and in (*12*), which report first-dose VE values of 30 to 33%, which increase to 61 to 67% for the fully vaccinated. The latter three studies were conducted in the United Kingdom, where the population may have had different seroprevalence rates; therefore, any comparisons here merit caution.

Specifically for West Bengal, our estimated VE against symptomatic infections (55.2%) is higher than those calculated for ChAdOx1 NCov-19 in (*27*), which find VE values of 46.2% for the first dose of ChAdOx1 NCov-19, and 63.1% for full vaccination (the B.1.617.2 variant was observed in 90% of the infected population in their case). For moderate-severe COVID, however, Thiruvengadam *et al*. (*27*) find a VE of 79.2% for the first dose only, and 81.5% for full vaccination, which are similar to our estimates.

While the RDD-based measures are valuable when the available data do not allow the use of alternative VE approaches (such as the TND) or if time/cost restrictions may prevent the use of randomized clinical trials, such trials remain the gold standard [e.g., (*28*)]. Randomization allows trials to avoid specific assumptions regarding attack rates, unlike the standard RDD here—or even the TND. The B-RDD also does not require such assumptions but still relies on local randomization, as explained earlier, and hence may not be valid for ages far away from the cutoff. This, however, is not a general issue for our framework—in other countries or contexts outside India, where multiple age-based cutoffs were used over time [e.g., (*21*, *29*)], RDD-based measures can be applied to each cutoff separately to generate VE measures that are relevant for populations around those different age cutoffs.

While the VE estimates that we report assume that a person has protection from vaccination 2 weeks after receiving their first dose, we also performed a sensitivity analysis using a 3-week lag, which does not materially change our estimates. In addition, we start our sample for the B-RDD measure on 10 April 2021, since vaccination status is more reliably recorded from that week onward in our data. Starting earlier could be problematic, because if some vaccinated patients in hospitals are recorded as unvaccinated, then our effectiveness estimate can be biased upward.

Furthermore, we measure the real-world effectiveness of vaccines—i.e., all consequences of being vaccinated, not just the medical effectiveness of the vaccine as measured in a clinical trial. For example, if the vaccinated change their behavior after vaccination such that, say, their exposure to COVID increases, this is also accounted for in our estimates. As a result, possible cross-country differences in such changes in behavior will also show up when these measures are applied in different countries.

Another limitation of our VE estimates is that they are calculated during the second wave in India, which was largely driven by the Delta variant. Therefore, these estimates may not apply to Omicron or other future variants. Nevertheless, such RDD-based measures could still be useful. For example, if vaccine makers develop new-generation vaccines that are modified for Omicron or other future variants (*30*), then estimates from developed countries (where the population already received a previous version of the vaccines) may again not be applicable to developing countries (where people might be getting the new version of the vaccine without being vaccinated earlier). If these developing countries use age-based eligibility for the new-generation vaccines (as with existing vaccines), then the RDD-based measures can allow them to calculate VE in the future.

Last, as we mentioned, many of the more standard VE techniques, such as case-control or TND, cannot be calculated using our data. This limits our ability to compare and contrast our VE numbers with those from such methods using the same dataset. However, these comparisons are possible in other countries, such as the United Kingdom, where such detailed information is available. We leave this for future research.

In conclusion, as the COVID pandemic moves on from largely vaccinated developed countries with good data to mostly unvaccinated poorer countries that lack such data, greater attention needs to be directed toward helping public health authorities in these countries to measure VE. Such measurement is potentially critical to public health decisions on whether to prioritize first vaccine doses to reach a wider population or fully vaccinate half of those people under the typical two-dose regimen. This task can take us beyond the data-intensive standard methods, as we demonstrate here using data from India’s lethal second COVID wave.

## METHODS

To present the RDD-based VE measures that we use, we first consider a single-dose vaccine regimen in a static framework and extend it to a two-dose vaccine in the Supplementary Materials. We use COVID hospitalizations, as a proxy for severe disease, to illustrate these measures. Readers interested in their application—rather than their underlying logic/derivation—can skip to the “Data” section.

### RDD-based setup for VE measures

Suppose that public health authorities decide that those aged 45 and above are eligible for vaccination, but anyone below that cutoff is not. Call the people just below (above) 45 the Left (Right) group and denote them *L* (*R*). Say, *L* could contain those aged 40 to 44, and *R* those aged 45 to 49. The choice of 45 as the cutoff is arbitrary and is typically not motivated by any medical reason. Hence, the fact that those on the Right are vaccinated but those on the Left are not is locally “as good as random”—i.e., Left and Right have similar distribution of characteristics, absent vaccines (*31*). Henceforth, we will consider the two groups to be identical for ease of exposition.

**Fig. 4.**
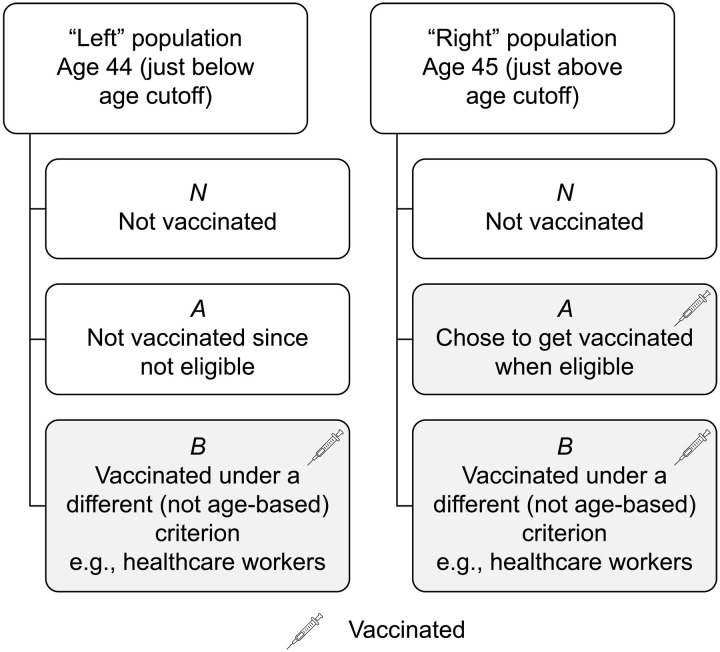
Subpopulations *A*, *B*, and *N* in our framework, with their respective vaccination status.

In the general case when some in the Right group are not vaccinated while some on the Left are (e.g., because they were health care workers), we can decompose each of the identical Left and Right groups into three subgroups, as illustrated in Fig. 4: (i) those who are vaccinated across both groups (denoted *B*); (ii) additional people vaccinated on the Right, who are not vaccinated on the Left, but would have been vaccinated had they been eligible (denoted by *A*); and (iii) those who are not vaccinated in either the Left or the Right group (denoted *N*).

Since we assume that these two groups are identical, the sizes of the subgroups *A*, *B*, and *N* as well as their respective attack rates α*_A_*, α*_B_*, and α*_N_* on the Left and the Right are the same. By definition, the attack rate for an unvaccinated person is the probability that they end up in hospital for COVID. For the vaccinated, it is the probability with which they would have been hospitalized with COVID had they not been vaccinated.

Let *e* be the VE rate, which here is the proportional reduction in COVID hospitalizations among the vaccinated relative to the unvaccinated. Then, the number of people hospitalized on the Left is equal to the number of those hospitalized from subgroups *N* and *A* (who are unvaccinated), plus those from subgroup *B* (who are vaccinated), i.e., *H_L_* = *N*α*_N_* + *A*α*_A_* + *B*α*_B_*(1 − *e*). Similarly, the number of people hospitalized on the Right is *H_R_* = *N*α*_N_* + *A*α*_A_*(1 − *e*) + *B*α*_B_*(1 − *e*). The difference between these two numbers is *H_L_* − *H_R_* = *A*α*_A_e*. We can recover *e* from this expression in two different ways, depending on the type of data available.

### Estimating VE with unknown individual vaccination status using standard RDD

From above, $\frac{H_{L}-H_{R}}{A}$ yields an unbiased and consistent estimate for α*_A_e*—the drop in hospitalization rate as a result of vaccinating set *A* on the Right. However, we still need a consistent estimator of α*_A_* to obtain a consistent estimator of *e* (the VE). This estimate is easily available if everyone on the Right, but none on the Left, are vaccinated (i.e., a sharp RDD)—then $\frac{H_{L}}{A}$ is a consistent and unbiased estimator of α*_A_*. When this is not the case (fuzzy RDD), we need further assumptions to obtain a consistent estimate of α*_A_*. For example, we could use observable covariates to identify a set of people on the Left who have attack rates very similar to those of the additionally vaccinated among the Right, and then use their attack rate to estimate α*_A_*. Alternatively, without any information on covariates, we can assume that α*_A_* = α*_B_* = α*_N_* = α (Assumption A1) and estimate *e* as

$$\begin{array}{c} H_{L}+\frac{B}{A}(H_{L}-H_{R})=N\alpha +A\alpha +B\alpha (1-e)+B\alpha e=(N+A+B)\alpha \\ \Rightarrow e=\frac{H_{L}-H_{R}}{A}\times \frac{1}{\alpha }=\frac{H_{L}-H_{R}}{A}(\frac{N+A+B}{H_{L}+\frac{B}{A}(H_{L}-H_{R})}) \end{array}$$

Let $X=\frac{H_{L}-H_{R}}{H_{L}}$ be the proportional drop in hospitalizations in Right relative to Left, $v_{L}=\frac{B}{B+A+N}$ be the vaccination rate for the Left group, and $v_{R}=\frac{B+A}{B+A+N}$ be the vaccination rate for the Right group. Then, the above formula yields

$$e=\frac{X}{(v_{R}-v_{L})+Xv_{L}}$$ (1)

### Estimating VE with known individual vaccination status using B-RDD

The B-RDD exactly recovers the number of people among those additionally vaccinated on the Right who would have been hospitalized had they not been vaccinated—given by *A*α*_A_*. This counterfactual number is not directly observable but can be recovered using a mathematical identity; it equals the sum of two quantities:

1) the number of additionally vaccinated people on the Right who do not end up in hospital because they are vaccinated—given by the difference in hospitalizations between the Left and the Right (*H_L_* − *H_R_*), and

2) the number of additionally vaccinated people on the Right who still end up in hospital although they are vaccinated—given by the difference in breakthrough hospitalizations between the Right and the Left (ψ*_R_* − ψ*_L_*). Note that this quantity is not used by the standard RDD.

Then, the identity is

$$A\alpha_{A}=(\psi_{R}-\psi_{L})+(H_{L}-H_{R})$$

Since *H_L_* − *H_R_* = *A*α*_A_e*, the identity implies

$$e=\frac{H_{L}-H_{R}}{\left(H_{L}-H_{R})+(\psi_{R}-\psi_{L}\right)}$$

Denoting $\mu_{L}=\frac{\psi_{L}}{H_{L}}$, $\mu_{R}=\frac{\psi_{R}}{H_{R}}$, and (as earlier) $X=\frac{H_{L}-H_{R}}{H_{L}}$, the B-RDD estimate of VE is

$$e=\frac{X}{X+\mu_{R}(1-X)-\mu_{L}}$$ (2)

This yields a consistent estimator for *e* because it is the ratio of two consistent estimators: $\frac{H_{L}-H_{R}}{A}$ for α*_A_e* and $\frac{\left(H_{L}-H_{R})+(\psi_{R}-\psi_{L}\right)}{A}$ for α*_A_*.

The B-RDD does not need to assume any equality of attack rates α*_A_* = α*_B_* = α*_N_*, unlike the standard RDD. It also does not depend on other assumptions needed for VE methods like cohort studies or a TND (see the Supplementary Materials).

Furthermore, even if not everyone with severe disease is hospitalized, and if our interest is really in measuring VE against severe disease, the B-RDD can still recover this VE of interest. This is because the B-RDD correctly matches the drop in hospitalization with the counterfactual number of people who would have been hospitalized absent vaccines—they are both measured from the set of people who are hospitalized when they get severe disease.

Beyond statistical advantages, the B-RDD can be calculated in certain situations where neither the standard RDD nor cohort studies/TND can be used. For example, if researchers do not have information regarding how many more 45- versus 44-year-olds are vaccinated in the population, the standard RDD (in particular, *v_R_* and *v_L_* in Eq. 1) cannot be calculated. However, if vaccination status is known for those in hospitals, the B-RDD (μ*_R_* and μ*_L_* in Eq. 2) can be calculated. Even if researchers know how many more 45- versus 44-year-olds are vaccinated in the population, this population (say the whole country) may be different from those who really have access to testing or hospitals (say, only the urban population), causing issues for the standard RDD. Furthermore, unlike the TND, the B-RDD does not require vaccination information for test-negative cases (which is not available, for example, in our hospitalization data from India). It also provides asymptotically unbiased VE estimates even when we only have data from just a subset of COVID hospitals that record information on vaccination status.

Last, we note that Eqs. 1 and 2 are derived assuming that the Left and Right groups have the same sizes and attack rates. In the Supplementary Materials, we show that even when they have different sizes and attack rates, the same formulas apply, with *H_R_* and *H_L_* in the definition of *X* scaled appropriately.

### Data

We obtain weekly COVID vaccinations data by district and age from CoWIN (COVID Vaccine Intelligence Network, India’s centralized vaccination records system). Our data for COVID tests and COVID hospitalizations, provided by the West Bengal Government’s Health and Family Welfare Department, contain all tests and all COVID-positive hospitalizations between 1 September 2020 and 21 May 2021. The testing data include the district and self-reported age of the tested persons, as well as the date of the test, test result, and specifics such as the cycle threshold value of the test. Specifically for our testing data, our estimates are based on PCR-positive tests that were performed in a population that was not randomly selected. Given that asymptomatic individuals were less likely to be tested, these estimates may be more representative of protection against symptomatic infections, which is how we interpret our results throughout. Our hospitalization data come from CPMS (the COVID Patient Management System), where hospitals in West Bengal are directed to upload in real-time COVID patient data. This dataset contains information on the age, district, date of admission, outcome (discharge or escalation, e.g., transfer to an ICU or death), and the vaccination status reported by the patient.

### Age heaping

**Fig. 5.**
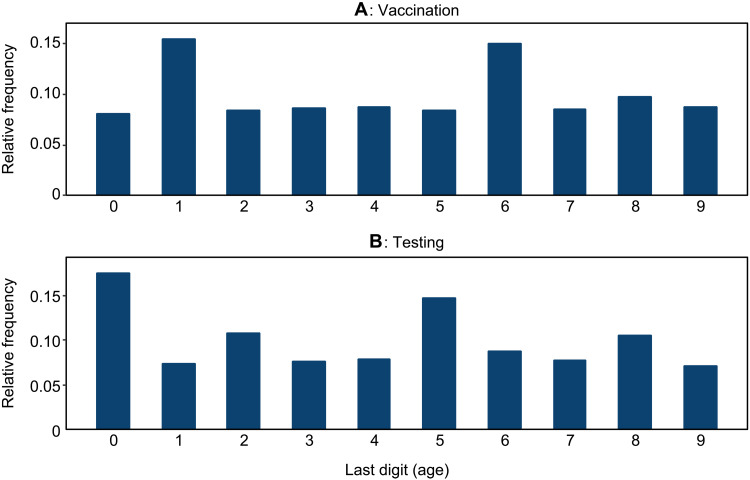
Age heaping. This figure shows histograms of the last digits of the ages reported in our datasets—in (**A**) for COVID vaccinations and in (**B**) for test results. Substantial heaping is observed at ages ending in one and six in (A) and at zero and five in (B).

While the formulas we provide are general and can be estimated in various contexts, the empirical methodology that we use is adapted to the specifics of our data. For example, one specific feature of our datasets is that certain ages are reported much more frequently than others (e.g., ages ending in zero or five in the COVID testing data, and those ending in one or six in the vaccination data, as shown in Fig. 5).

Such age heaping is a well-recognized data issue in different contexts [e.g., (*32*)] and can be especially problematic in developing countries [e.g., (*33*)]. It is a particularly relevant issue for the RDD approach, which compares observations around an age cutoff—e.g., 44-year-olds versus 45-year-olds—since people who report their age as, say, a multiple of five could differ in essential ways from those who do not, and then our comparisons could be confounded by such differences. For example, if a person had reported a round-number age in the national identification card (called Aadhaar card in India) used for vaccinations but reported their true age to doctors at the hospital, RDD estimates would be affected by the resultant noise. Following (*34*), we discard the ages that are most susceptible to such heaping—i.e., those in our sample that end in zero, one, five, and six. The logic here is that people who do not round their ages in one dataset are also unlikely to do so in another—resulting in the ages being consistently captured across the same unit. We report throughout results based on aggregates of three consecutive ages (e.g., 42, 43, 44 or 47, 48, 49).

### Calculating VE in our sample

To reduce noise, we use appropriate weighted averages of the inputs in Eqs. 1 and 2 over the entire sample to calculate one VE estimate. The weights reflect the fact that our numbers are likely to be noisier in weeks with fewer underlying cases (e.g., a drop from 2 to 1 hospitalized in a certain week is likely to be less informative than a drop from 20,000 to 10,000 in another, although they are the same in percentage terms). To account for such noise, the observations for each week are weighted by the total number of hospitalizations in that week.

Calculating the VE measure from Eq. 1 (i.e., when the vaccination status is not known) requires vaccination rates (*v_L_* and *v_R_*) in the population, as well as the percentage drop in COVID outcomes denoted by *X*. Since we assume that it takes 2 weeks for vaccines to take effect, we use vaccination data preceding by 2 weeks the data used in calculating *X*—vaccinations for those aged 45 and above started on 1 March 2021, so our sample for *X* here starts on 13 March. In turn, the VE measure in Eq. 2 requires data on the percentage of vaccinated among those in hospital (μ*_L_* and μ*_R_*). Because these data are mostly missing before 17 April 2021, in this case, we drop the observations before this date.

To account for possible age-related differences in outcomes that are not due to vaccinations, we use data from a prevaccination period to adjust *X* (see the Supplementary Materials). For example, with COVID infections, we calculate $X=1-\frac{I_{\text{post},R}}{I_{\text{pre},R}}/\frac{I_{\text{post},L}}{I_{\text{pre},L}}$, where *I* is the average weekly number of symptomatic COVID infections in the pre- (post)vaccination period among those in the Left or Right groups, which comprise those aged 42 to 44 or 47 to 49 years, respectively. The *X* variables for other outcomes, e.g., high-viral load infections, hospitalizations, and escalations/deaths, are calculated analogously.

When more granular data—e.g., data on outcomes at the district level within a state—are available, we can adopt a regression-based approach to account also for potential differences across these districts [e.g., (*35*)] (see the Supplementary Materials for such estimates).

### Construction of confidence intervals

We generate confidence intervals via a bootstrap procedure. To illustrate this procedure with respect to hospitalizations (bootstraps are done analogously for all other outcomes), we start with our dataset containing 141,800 hospitalizations. For each draw of the bootstrap, we sample with replacement as many observations from this dataset and use that sample for our calculations. We repeat the procedure 1000 times and calculate the effectiveness measure as per Eqs. 1 and 2 each time to obtain an empirical distribution of these 1000 VE measures. The reported 95% confidence intervals show the 2.5 and 97.5 percentiles of this empirical distribution.

### Estimating VE for a two-dose vaccine

When a vaccine requires two doses, as in the case of COVID, we next show how the expressions in Eqs. 1 and 2 can provide an estimate of a weighted average of the effectiveness of the two doses. As earlier, we have two groups, denoted Left and Right, that are identical in all respects (assumed for ease of exposition), except that the Right group includes more people who are eligible for vaccination than the Left do. We assume that there is no one who is eligible for one vaccine dose on the Left but is eligible for two doses on the Right. This is true for our empirical implementation, in which we stopped the sample period before everyone below the 45-year threshold became eligible. Some people younger than 45 years of age were eligible for vaccination (e.g., health care workers). Even in this set, no one would be eligible for only one dose if they were below the age of 45 years but one would be eligible for two doses if they were above that age because the age threshold was not relevant for determining eligibility in this group.

With this assumption, the two identical Left and Right groups can be broken down into five subgroups: (i) vaccinated with one dose across both groups, denoted *B*_1_; (ii) vaccinated with two doses across both groups, *B*_2_; (iii) unvaccinated in the Left but vaccinated with one dose in the Right, *A*_1_; (iv) unvaccinated in the Left but vaccinated with two doses in the Right, *A*_2_; and (v) unvaccinated in both the Left and Right groups, *N*. Note that the earlier subgroups *A*, *B*, and *N*, if defined based on at least one vaccine dose, have a correspondence with the subgroups here. Subgroup *B* is divided into *B*_1_ and *B*_2_, and subgroup *A* is divided into *A*_1_ and *A*_2_. Subgroup *N* is the same as earlier. As we did previously, we will use these labels to represent the sizes of the respective subgroups. Therefore, *A* = *A*_1_ + *A*_2_ and *B* = *B*_1_ + *B*_2_. We assume that the effectiveness of the first and second doses of the vaccine is *e*_1_ and *e*_2_, respectively.

First, we evaluate the formula for *e* in Eq. 2. We assume that the attack rate for *A*_1_ is α_*A*_1__ and that for *A*_2_ is α_*A*_2__. Therefore, *A*α*_A_* = *A*_1_α_*A*_1__ + *A*_2_α_*A*_2__. Also, *H_L_* − *H_R_* = *A*_1_α_*A*_1__*e*_1_ + *A*_2_α_*A*_2__*e*_2_, and ψ*_R_* − ψ*_L_* = *A*_1_α_*A*_1__(1 − *e*_1_) + *A*_2_α_*A*_2__(1 − *e*_2_). Therefore

$$\begin{array}{c} \frac{X}{X+\mu_{R}(1-X)-\mu_{L}}=\frac{H_{L}-H_{R}}{\left(H_{L}-H_{R})+(\psi_{R}-\psi_{L}\right)}=(\frac{A_{1}\alpha_{A_{1}}}{A\alpha_{A}})e_{1}+\\ (\frac{A_{2}\alpha_{A_{2}}}{A\alpha_{A}})e_{2} \end{array}$$

which is a weighted average of the effectiveness rates of the first and second doses, where the weights are proportional to the expected numbers of people—from subgroups *A*_1_ and *A*_2_—who would have been hospitalized if they were unvaccinated.

We now evaluate the formula for *e* in Eq. 1 under the assumption that all five subgroups have equal attack rates α.

$$\begin{array}{c} \frac{X}{(v_{R}-v_{L})+{Xv}_{L}}=\frac{\left(A_{1}e_{1}+A_{2}e_{2})(B+A+N\right)}{A(N+A+B_{1}(1-e_{1})+B_{2}(1-e_{2}))+(A_{1}e_{1}+A_{2}e_{2})B}\\ =(\frac{A_{1}}{A}e_{1}+\frac{A_{2}}{A}e_{2})(\frac{1}{1+v_{L}(e_{2}-e_{1})(\frac{A_{2}}{A}-\frac{B_{2}}{B})}) \end{array}$$

This is very close to the weighted average of efficacy rates *e*_1_ and *e*_2_, with weights proportional to *A*_1_ and *A*_2_, if either *v_L_* is small (as is the case in our sample), or *e*_2_ is close to *e*_1_, or $\frac{A_{2}}{A}$ is close to *cB*_2_*B*.

We can use this expression to generate bounds for VE, under the assumption that the effectiveness of two doses is at least as high as that of one dose. Note that our RDD estimates for VE are weighted averages of the one- and two-dose efficiency numbers, given that *v_L_* is small in our sample. The weights *A*_1_ (*A*_2_), as mentioned, are the number of additional people to the right of the 45-year cutoff who were vaccinated with one (two) vaccine dose(s), relative to those on the left. These numbers are the averages over our sample since 1 March, separately for the first and second doses, calculated using data from CoWin.

Under these assumptions, the upper bound for the first dose VE is obtained assuming that the VE values for one and two doses are equal. A lower bound is obtained when we assign the VE for two doses its maximum value of 100%.

## Supplementary Materials

### Other Supplementary Material for this manuscript includes the following:

Spreadsheet S1
