# Supplementary material for: Measuring vaccine effectiveness from limited public health datasets: Framework and estimates from India’s second COVID wave
Source: Sci Adv. 2022 May 6;8(18):eabn4274. doi: 10.1126/sciadv.abn4274 (PMC9075799; doi:10.1126/sciadv.abn4274)
Supplement: Supplementary file 1 — Supplementary Text Fig. S1 Tables S1 and S2 [file sciadv.abn4274_sm.pdf]

Science Advances
AAAS

Supplementary Materials for

Measuring vaccine effectiveness from limited public health datasets: Framework and estimates from India's second COVID wave

Abhiroop Mukherjee, George Panayotov, Rik Sen, Harsha Dutta, Pulak Ghosh*

*Corresponding author. Email: pulak.ghosh@iimb.ac.in

Published 6 May 2022, *Sci. Adv.* **8**, eabn4274 (2022)
DOI: 10.1126/sciadv.abn4274

The PDF file includes:

Supplementary Text
Fig. S1
Tables S1 and S2
Legend for spreadsheet S1

Other Supplementary Material for this manuscript includes the following:

Spreadsheet S1

Supplementary Text

Comparison to Standard VE Measures

Let S be the population size and v be the vaccination rate ($0 \leq v \leq 1$). Let α_V be the attack rate for the vaccinated people, and α_N be the attack rate for the unvaccinated population ($0 \leq \alpha_V, \alpha_N \leq 1$).

Under the setup in the main text, the expected number of people hospitalized for COVID is the number of unvaccinated people hospitalized, plus the number of vaccinated people hospitalized, i.e., $H = S(1-v)\alpha_N + Sv\alpha_V(1-e)$. Then the (expected) fraction of the hospitalized who are vaccinated is $h = Sv\alpha_V(1-e)/H$.

$$1 - e = \left(\frac{h}{1-h}\right) / \left(\frac{v}{1-v}\right) \times \frac{\alpha_N}{\alpha_V}$$

If we observe v and h and assume that $\alpha_V = \alpha_N$ (Assumption A2), we can estimate VE as:

$$e = 1 - \left(\frac{h}{1-h}\right) / \left(\frac{v}{1-v}\right) = \frac{v-h}{v-vh} \tag{S1}$$

A cohort study measures VE in this spirit, using, for example, the incidence rate ratios of outcomes among the vaccinated relative to the unvaccinated (e.g., (*29*)). When applying this method, researchers typically try to ensure that the vaccinated and unvaccinated are as similar as possible, so that the assumption $\alpha_V = \alpha_N$ is more reasonable.

A TND, on the other hand, allows for the vaccinated and unvaccinated to be different (e.g., in terms of their access to hospitals). It then tries to adjust for this difference using data for those who presented COVID-like symptoms and were hospitalized, but then tested negative for COVID (e.g., they were diagnosed with some non-COVID respiratory infection like the flu). If β_V and β_N are the attack rates for the flu among the COVID-vaccinated and unvaccinated, and assuming that COVID vaccination has no effect on the flu attack rates, the fraction of vaccinated among those hospitalized for the flu is: $h_\beta = \frac{Sv\beta_V}{S(1-v)\beta_N + Sv\beta_V}$. Assuming that $\frac{\alpha_V}{\alpha_N} = \frac{\beta_V}{\beta_N}$ (Assumption A3), we get:

$$e_{TND} = 1 - \left(\frac{h}{1-h}\right) / \left(\frac{h_\beta}{1-h_\beta}\right) = \frac{h_\beta - h}{h_\beta - h_\beta h} \tag{S2}$$

Two critical issues hinder the application of such standard VE measures to public health datasets in developing countries (*7*):

1. Information on vaccination status among cases (and/or for all those who test negative),

is not available in many developing countries, making these measures impossible to calculate.

2. Even when such data are available, these measures crucially rely on assumptions about attack rates across the vaccinated and unvaccinated populations, as in Assumptions A2 and A3 above. Most studies acknowledge that there could be unobservable differences between these populations which, by definition, are impossible to control for, thus creating concerns regarding these assumptions.

The measures described in the *Methods* section of the paper avoid these shortcomings.

Adjusting for different sizes and attack rates across the *Left* and *Right* groups

The formula derivations in *Methods* assumed that the *Left* and *Right* groups have the same sizes; now we relax this assumption. Hence we have to define the sub-groups B, A, and N separately on the *Left* and *Right* (B_L and B_R, A_L and A_R, and N_L and N_R). We assume that although the group sizes are different, the proportions of these three sub-groups remain the same, i.e., $A_L/A_R = B_L/B_R = N_L/N_R = L/R$, where $L = A_L + B_L + N_L$ and $R = A_R + B_R + N_R$ denote the sizes of the two groups (e.g., the same percentage of the people on *Left* and *Right* are health-care workers). Then the vaccination rate for *Left* is $v_L = B_L/L$, and the difference in vaccination rates is $v_R - v_L = A_L/L = A_R/R$.

We denote the attack rates for these six sub-groups by α_{B_L}, α_{B_R}, α_{A_L}, α_{A_R}, α_{N_L}, and α_{N_R}, respectively. The number of people hospitalized among the *Left* and *Right* are now given by $H_L = N_L\alpha_{N_L} + A_L\alpha_{A_L} + B_L\alpha_{B_L}(1-e)$ and $H_R = N_R\alpha_{N_R} + A_R\alpha_{A_R}(1-e) + B_R\alpha_{B_R}(1-e)$.

We now exploit information from a pre-vaccination period. During this period we observe the hospitalization numbers of the *Left* and *Right* groups – H_L^{pre} and H_R^{pre}. Let α_L^{pre} and α_R^{pre} are the attack rates in the pre-vaccination period. Then, $H_L^{pre} = (A_L+B_L+N_L)\alpha_L^{pre} = L\alpha_L^{pre}$ and $H_R^{pre} = (A_R + B_R + N_R)\alpha_R^{pre} = R\alpha_R^{pre}$.

Recall that the standard RDD (equation (1)) assumed the equality of within-group attack rates (Assumption A1), which here becomes $\alpha_{A_L} = \alpha_{B_L} = \alpha_{N_L} = \alpha_L$ and $\alpha_{A_R} = \alpha_{B_R} = \alpha_{N_R} = \alpha_R$ in the post period, and $\alpha_{A_L}^{pre} = \alpha_{B_L}^{pre} = \alpha_{N_L}^{pre} = \alpha_L^{pre}$ and $\alpha_{A_R}^{pre} = \alpha_{B_R}^{pre} = \alpha_{N_R}^{pre} = \alpha_R^{pre}$ in the pre-period. Therefore

$$\frac{H_L}{A_L\alpha_L} - \frac{H_R}{A_R\alpha_R} = e$$

We further assume stability of relative risks across the two groups over different periods, i.e., the ratio of attack rates across *Left* and *Right* in the pre-vaccination period is the same as the ratio in the post-vaccination period – $\alpha_L^{pre}/\alpha_R^{pre} = \alpha_L/\alpha_R$ (Assumption A4).

Denoting $X_{adj} = 1 - \frac{H_R/H_R^{pre}}{H_L/H_L^{pre}}$, we can verify that

$$e = \frac{X_{adj}}{(v_R - v_L) + X_{adj} v_L} \tag{S3}$$

Note that this is the same expression as equation (1) with X replaced by X_{adj}.

Equation (S3) is derived from the expressions written in this section as follows

$$H_L = L\alpha_L - B_L\alpha_L e = (L - B_L e)\alpha_L \quad \text{and} \quad v_L = B_L/L \quad \text{and} \quad v_R - v_L = A_L/L$$

$$\frac{H_L^{pre}}{H_R^{pre}} = \frac{L}{R}\frac{\alpha_L^{pre}}{\alpha_R^{pre}} = \frac{A_L}{A_R}\frac{\alpha_L}{\alpha_R} \implies A_R\alpha_R = \frac{H_R^{pre}}{H_L^{pre}} A_L\alpha_L$$

$$e = \frac{H_L}{A_L\alpha_L} - \frac{H_R}{A_R\alpha_R} \implies \frac{eA_L\alpha_L}{H_L} = 1 - \frac{H_R/H_R^{pre}}{H_L/H_L^{pre}} = X_{adj} \implies \frac{eA_L\alpha_L}{(L - B_L e)\alpha_L} = X_{adj}$$

$$\implies e = X_{adj}\left(\frac{L}{A_L} - \frac{B_L e}{A_L}\right) \implies e = \frac{X_{adj}}{A_L/L + X_{adj}B_L/L} = \frac{X_{adj}}{(v_R - v_L) + v_L X_{adj}}$$

Next we move to the B-RDD measure, which does not need to assume equality of the attack rates within each age group (i.e, Assumption A1). Here, instead of requiring A1 and A4, we assume $\alpha_L^{pre}/\alpha_R^{pre} = \alpha_{A_L}/\alpha_{A_R}$, which is a weaker assumption. (It holds if, for example, assumption A4 holds and $\alpha_{A_L}/\alpha_{A_R} = \alpha_{B_L}/\alpha_{B_R} = \alpha_{N_L}/\alpha_{N_R}$ in both the pre- and the post-vaccination periods. In contrast, A1 requires equality of the attack rates across different sub-groups – i.e., $\alpha_{A_L} = \alpha_{B_L} = \alpha_{N_L}$ and $\alpha_{A_R} = \alpha_{B_R} = \alpha_{N_R}$ in the two periods). The expressions for ψ_L and ψ_R, i.e., the number of vaccinated people among the hospitalized for the *Left* and *Right*, change to $\psi_L = B_L\alpha_{B_L}(1-e)$, and $\psi_R = A_R\alpha_{A_R}(1-e) + B_R\alpha_{B_R}(1-e)$. As earlier, $\mu_L = \frac{\psi_L}{H_L}$ and $\mu_R = \frac{\psi_R}{H_R}$.

It follows that

$$\left(\psi_R \frac{L\alpha_{A_L}}{R\alpha_{A_R}} - \psi_L\right) + \left(H_L - H_R\frac{L\alpha_{A_L}}{R\alpha_{A_R}}\right) = A_L\alpha_{A_L}, \text{ and}$$

$$H_L - H_R\frac{L\alpha_{A_L}}{R\alpha_{A_R}} = A_L\alpha_{A_L} e$$

Taking the ratio of these two expressions, we get

$$e = \frac{H_L - H_R\frac{L\alpha_{A_L}}{R\alpha_{A_R}}}{\left(\psi_R\frac{L\alpha_{A_L}}{R\alpha_{A_R}} - \psi_L\right) + \left(H_L - H_R\frac{L\alpha_{A_L}}{R\alpha_{A_R}}\right)} = \frac{1 - \frac{H_R}{H_L}\frac{L\alpha_{A_L}}{R\alpha_{A_R}}}{\left(\frac{\psi_R}{H_R}\frac{H_R}{H_L}\frac{L\alpha_{A_L}}{R\alpha_{A_R}} - \frac{\psi_L}{H_L}\right) + \left(1 - \frac{H_R}{H_L}\frac{L\alpha_{A_L}}{R\alpha_{A_R}}\right)}$$

Defining X_{adj} as above, the VE estimate is given by

$$e = \frac{X_{adj}}{X_{adj} + \mu_R(1 - X_{adj}) - \mu_L} \tag{S4}$$

Sensitivity Analysis

Regression-based estimates using more granular data

When district level data is available, a regression-based approach we can be adopted to account for potential differences across these districts. These differences in COVID patterns across different districts (e.g., due to the concentration of hospitals and testing facilities) could also vary over time (e.g., some districts affected more by the first wave than the second wave).

To account for such heterogeneity, we start by including district-time fixed effects in our regressions. These ensure that any patterns of infections or testing that varied across districts – but affected all ages at the same time – are netted out of our estimates. However, even after doing so, the percentage drop estimates for X in equations (1) and (2) are likely to be noisier when the number of underlying cases is lower. To account for such noise, we estimate weighted least squares regressions, weighting each district-week-age observation by the total number of hospitalizations in that district-week, similar to our approach in the *Methods* section.

To formally verify that vaccinations indeed showed a jump at the eligibility cutoff age of 45 years, we start with the following regression, utilizing the data for all available age groups:

$$Y_{d,a,t} = \beta_0 + \sum \beta_a \text{Age}_a + \sigma_{d,t} + \epsilon_{d,a,t} \tag{S5}$$

where $Y_{d,a,t}$ is the proportion of vaccinated people in district d for age group a at time t, $\sigma_{d,t}$ are district-time fixed effects as explained above, and Age_a are age dummies.

The β_a estimates are the differences in the vaccination rates between each age group and one reference age group (for our regressions this is the group of 22-24 year olds, but our results are not sensitive to this choice). The β_a for the age-group immediately to the right (left) of 45 years delivers v_R (v_L) in the VE measure in equation (1) in the paper. We also

estimate a similar specification when we know the vaccination status among the hospitalized, and then Y_t is the proportion of vaccinated people among those in an age group and district *who are hospitalized* at time t. In that specification, analogous β_a coefficients around the cutoff deliver μ_R and μ_L in the VE measure in equation (2) in the paper.

The next step is to assess whether there are corresponding discontinuous drops in outcomes at the same cutoff. Since age cutoff-based vaccinations started in March, here we can use the difference in the age structure of outcomes between the pre- and post-vaccination periods to distil out the effect of vaccinations at the cutoff. We do so by running the following regression:

$$Y_{d,a,t} = \gamma_0 + \sum \gamma_a \text{Age}_a \times \text{Post}_t + \sigma_{d,t} + \delta_{a,t} + \epsilon_{d,a,t} \tag{S6}$$

where $Y_{d,a,t}$ is the log(1+number of people with certain outcome), and $Post_t$ is an indicator for the post-vaccination period (dummy variable), which takes the value of 1 after 13 March 2021 and 0 otherwise, $\sigma_{d,t}$ are district-time fixed effects, $\delta_{a,t}$ are district-age fixed effects that control for time-invariant and age-specific characteristics within each district. The coefficients γ_a measure the incremental effect at each age coming from the post-vaccination period. The drop in the γ_a coefficients around the cutoff delivers the X in equations (1) and (2) in the paper. Results from this regression-based methodology is presented in Table S1, which is analogous to Table 1 in the paper.

Measuring VE starting three weeks post-vaccination

In Table S2 we present VE measures similar to those in Table 1 in the paper, assuming that COVID vaccines provide protection after three weeks following the first dose (rather than two weeks as in the paper). The table format is as in Table 1 in the paper.

Placebo results from the pre-vaccination period

Figure S1 shows placebo results with a "counterfactual" post-vaccination period (10 November 2020 - 10 January 2021), which is similar in duration to the one we use in our main results, but ends before vaccinations begin in West Bengal. Unlike in Figure 4 in the paper, the plots in Figure S1 do not show a downward jump for any of the four displayed outcomes.

Table S1: Vaccine Effectiveness results: All numbers in this table are in percent. v_L, v_R, μ_L, μ_R and X are the inputs to the two VE formulas, obtained as per equations (S5) and (S6). Also shown are VE's and 95% bootstrap confidence intervals (CI). In Panel A, the VE's are calculated with the standard RDD, as per equation (1), and in Panel B they are calculated with B-RDD, as per equation (2). The last five columns of the table show the same statistics as in the first five, but now using values *at* the 45-years age cutoff of smoothing splines fitted to the left and right of this cutoff, analogous to those in Figure 3 in the paper.

	Local randomization					Continuity-based framework				
Panel A.	v_L	v_R	X	VE	95% CI	v_L	v_R	X	VE	95% CI
RTPCR-positive COVID tests	3.7	23.5	12.4	60.9	[46.5, 74.4]	3.8	16.9	8.5	61.5	[35.9, 87.0]
Tests with CT value $<$ 25	3.7	23.5	14.5	71.3	[37.7, 105.3]	3.8	16.9	11.1	79.1	[15.3, 141.5]
Panel B.	μ_L	μ_R	X	VE	95% CI	μ_L	μ_R	X	VE	95% CI
Hospitalizations	-1.6	2.2	14.9	80.9	[66.1, 89.5]	-1.8	1.5	14.4	83.1	[59.4, 93.0]
Escalations/deaths	-1.9	1.0	13.5	83.1	[19.1, 102.3]	-1.7	0.0	10.1	85.3	[35.9, 178.2]

Table S2: VE with three-week lag after vaccination: This table shows results analogous to those in Table 1 in the paper, but assuming a three-week interval before vaccines become effective.

	Age groups around the cutoff					Extrapolation around the cutoff				
Panel A.	v_L	v_R	X	VE	95% CI	v_L	v_R	X	VE	95% CI
RTPCR-positive COVID tests	4.9	22.5	10.9	60.1	[50.1, 71.5]	5.1	16.9	7.6	62.7	[42.4, 85.1]
Tests with CT $<$ 25	4.9	22.5	12.5	68.8	[39.2, 100.01]	5.1	16.9	9.5	77.2	[22.3, 138.6]
Panel B.	μ_L	μ_R	X	VE	95% CI	μ_L	μ_R	X	VE	95% CI
Hospitalizations	1.9	5.8	16.6	84.6	[74.9, 90.6]	1.7	4.8	16.0	87.3	[74.2, 94.8]
Escalations/deaths	1.1	4.3	19.9	89.4	[66.8, 98.3]	1.1	3.0	13.4	89.9	[-8.0, 164.9]

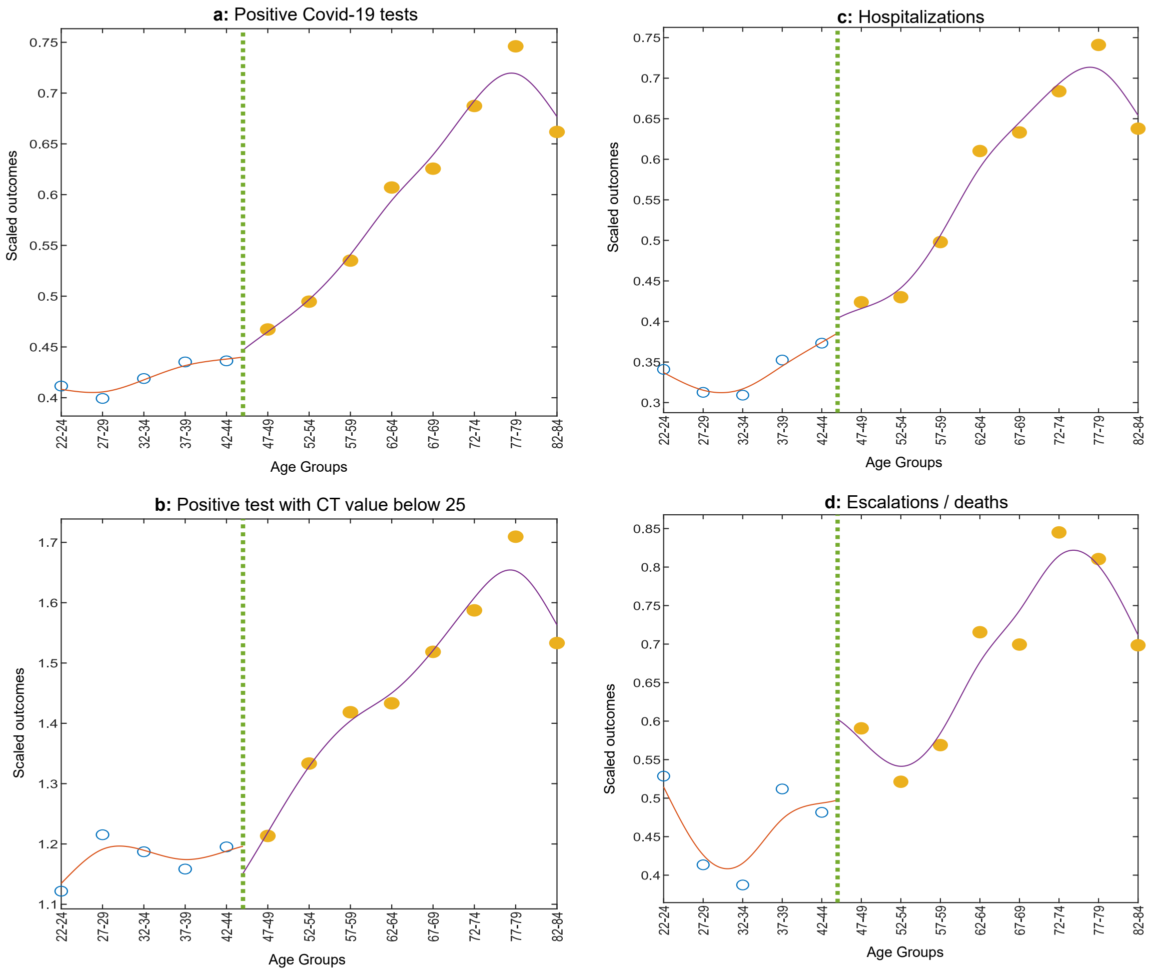

Figure S1: Placebo results from the pre-vaccination period: In the format of Figure 3 in the paper, this figure shows outcomes for various age groups. However, the “pre-” and “post-vaccination” periods are defined here as 5 September - 9 November 2020 and 10 November 2020 - 10 January 2021.

Spreadsheet S1. Simple implementation of RDD-based VE measures.
